# Type 5 phosphodiesterase regulates glioblastoma multiforme aggressiveness and clinical outcome

**DOI:** 10.18632/oncotarget.14656

**Published:** 2017-01-14

**Authors:** Valeriana Cesarini, Maurizio Martini, Lucia Ricci Vitiani, Giovanni Luca Gravina, Silvia Di Agostino, Grazia Graziani, Quintino Giorgio D'Alessandris, Roberto Pallini, Luigi M Larocca, Pellegrino Rossi, Emmanuele A Jannini, Susanna Dolci

**Affiliations:** ^1^ Department of Biomedicine and Prevention, University of Rome Tor Vergata, Rome, Italy; ^2^ Institute of Pathological Anatomy, Catholic University of Rome, Rome, Italy; ^3^ Department of Hematology, Oncology and Molecular Medicine, Istituto Superiore di Sanità (ISS), Rome, Italy; ^4^ Department of Biotechnological and Applied Clinical Sciences, Laboratory of Radiobiology, University of L'Aquila, L'Aquila, Italy; ^5^ Regina Elena National Cancer Institute-IFO, Oncogenomic and Epigenetic Unit, Rome, Italy; ^6^ Department of Systems Medicine, University of Rome Tor Vergata, Rome, Italy; ^7^ Department of Neurosurgery, Catholic University of Rome, Rome, Italy

**Keywords:** PDE5, PARP, glioblastoma, MYPT, MMP2

## Abstract

Expression of type 5 phosphodiesterase (PDE5), a cGMP-specific hydrolytic enzyme, is frequently altered in human cancer, but its specific role in tumorigenesis remains controversial. Herein, by analyzing a cohort of 69 patients affected by glioblastoma multiforme (GBM) who underwent chemo- and radiotherapy after surgical resection of the tumor, we found that PDE5 was strongly expressed in cancer cells in about 50% of the patients. Retrospective analysis indicated that high PDE5 expression in GBM cells significantly correlated with longer overall survival of patients. Furthermore, silencing of endogenous PDE5 by short hairpin lentiviral transduction (sh-PDE5) in the T98G GBM cell line induced activation of an invasive phenotype. Similarly, pharmacological inhibition of PDE5 activity strongly enhanced cell motility and invasiveness in T98G cells. This invasive phenotype was accompanied by increased secretion of metallo-proteinase 2 (MMP-2) and activation of protein kinase G (PKG). Moreover, PDE5 silencing markedly enhanced DNA damage repair and cell survival following irradiation. The enhanced radio-resistance of sh-PDE5 GBM cells was mediated by an increase of poly(ADP-ribosyl)ation (PARylation) of cellular proteins and could be counteracted by poly(ADP-ribose) polymerase (PARP) inhibitors. Conversely, PDE5 overexpression in PDE5-negative U87G cells significantly reduced MMP-2 secretion, inhibited their invasive potential and interfered with DNA damage repair and cell survival following irradiation. These studies identify PDE5 as a favorable prognostic marker for GBM, which negatively affects cell invasiveness and survival to ionizing radiation. Moreover, our work highlights the therapeutic potential of targeting PKG and/or PARP activity in this currently incurable subset of brain cancers.

## INTRODUCTION

Astrocytic brain tumors are divided into low-grade and high-grade astrocytomas on the basis of specific parameters established by the WHO (presence or absence of nuclear atypia, mitosis, necrosis and angiogenesis). Low-grade tumors comprise pilocytic astrocytomas (grade I), fibrillary, protoplasmic and gemistocytic astrocytomas (grade II), whereas anaplastic astrocytoma (grade III) and glioblastoma multiforme (GBM; grade IV) are classified as high-grade tumors [1, 2]. Although the cell of origin for glioma is still under debate, good candidates are NSCs or NSC-derived astrocytes or oligodendrocyte precursor cells (OPCs) [3]. GBM is the most common and fatal type of primary brain tumors, with annual incidence of about 3.19 cases per 100,000 in the population. Most patients survive approximately 1 year, and only 5% live for more than 5 years [1, 4]. GBM develops de novo by accumulation of genetic alterations in healthy cells [1], however, accumulation of additional genetic lesions in low-grade tumors can also determine their evolution to secondary GBM. Standard treatment for GBM consists of maximal surgical resection followed by 6 weeks of radiotherapy, plus concomitant adjuvant chemotherapy with temozolomide (TMZ) [5]. Prognostic factors for overall survival (OS) include patient status, tumor grade, and type of oncological treatment [6]. Methylation status of the O(6)-methylguanine-DNA methyltransferase (*MGMT*) gene promoter [7], mutations in the isocitrate dehydrogenase 1 (*IDH1*) and 2 (*IDH2*) genes [8] and epidermal growth factor receptor (EGFR) amplification or truncation (EGFRvIII) are considered additional molecular markers of prognostic value [9].

PDE5 is a cGMP-specific hydrolytic enzyme that has been extensively studied as it is the target of widely prescribed erectile dysfunction drugs (sildenafil, vardenafil, tadalafil and avanafil). The *PDE5A* gene maps at human chromosome 4q26 and encodes three alternatively spliced isoforms differing in their first exon [10, 11]. PDE5A transcripts are expressed in several tissues and cell types, including smooth muscle, cerebellum, retina and platelets [10]. Within the brain, PDE5 has been reported to be expressed within the hippocampus cortex, basal ganglia, cerebellum and in neural stem cells (NSC) [12–14]. Conflicting data have been reported on the role of PDE5 in cancer. PDE5 is expressed in several tumor types, such as breast, colon, bladder and lung carcinomas [15] and its inhibition was shown to enhance the cytotoxic effects of chemotherapy in prostate cancer and in murine and human brain tumor models [16–18]. By contrast, a negative correlation between PDE5 expression and tumor invasiveness was observed in metastatic melanoma [19, 20], a cancer type of neuro-ectodermal origin. In particular, it was shown that BRN2, a V600EBRAF activated target, represses PDE5 expression, thus increasing spreading of metastases. In support of this finding, it was reported that patients treated with sildenafil exhibited a higher risk of developing melanoma than untreated subjects [21]. PDE-mediated hydrolysis of intracellular cGMP is balanced by guanylate cyclase enzymes (GCs). Increased cGMP levels activate the PKGs and their downstream effectors [22]. Interestingly, the nitric oxide (NO)/cGMP/PKG system has been proposed to be involved in GBM stem cell expansion [23] and high levels of cGMP, as well as treatment with sildenafil, strongly enhance mouse GBM cancer stem cell phenotype *in vitro* and their tumorigenic potential *in vivo* [23]. In this study, we explored the prognostic value of PDE5 in GBM patients and investigated whether modulation of PDE5 function influences GBM cell invasiveness and resistance to radiotherapy.

## RESULTS

### PDE5 expression positively correlates with overall survival rates in primary GBMs

**Figure 1:**
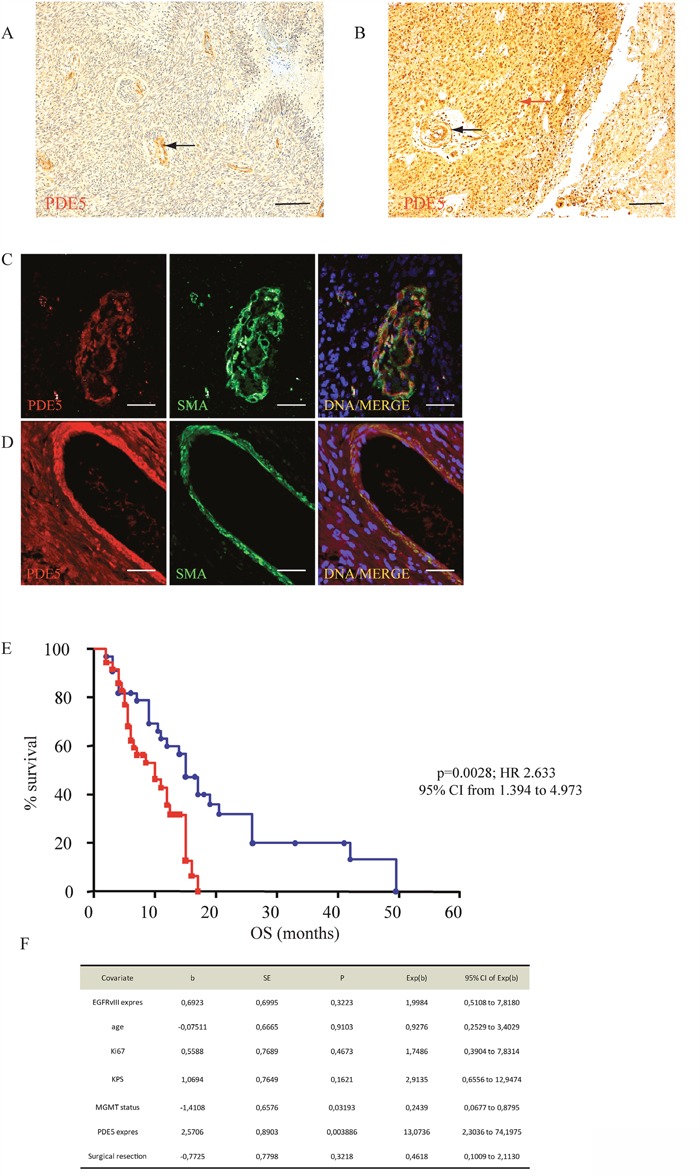
PDE5 expression correlates with OS in GBM **A**. PDE5 negative and **B**. PDE5 positive GBM samples, as revealed by immuno-histochemistry. Red arrow points to positive cancer cells, black arrows point to positive vascular smooth muscle cells. Scale bar=50μm, 100X magnification. PDE5 and smooth muscle actin (SMA) expression in smooth muscle cells in a PDE5 negative **C**. and in a PDE5 positive **D**. GBM sample. Scale bar=10μm. **E**. Kaplan–Meier curves representing the OS of 69 patients affected by GBM, (PDE5 positive, blue circles; PDE5 negative, red squares) after TMZ and X-ray treatments. **F**. The hazard ratios and 95% confidence intervals were calculated using a multivariable-adjusted Cox model, and the *p* values were calculated using a two-sided log-rank test.

To assess the value of PDE5 expression as molecular prognostic marker for GBM, we analyzed its levels in tumor sections obtained from 69 patients who underwent radiotherapy and TMZ treatment following surgical resection. In about 50% of these patients we found a strong PDE5 immuno-reactivity (score 4-9) in cancer cells. With the exception of vascular smooth muscle cells, PDE5 was not expressed in the unaffected surrounding tissue. The remaining 50% of cases showed low or no PDE5 immuno-staining in the tumor, while positivity was still found in the vascular structures (Figure 1A-1D). Next, we examined the associations of PDE5 expression with the clinical outcome of patients followed for a median period of 40 months (range= 2–50 months). Retrospective data analysis showed that high PDE5 expression in tumor cells strongly correlated with an increased OS (15 months *vs* 10 months, p=0,0028; Figure 1E). Multivariate analyses including EGFRvIII expression, age, KI67 index, KPS, *MGMT* status (Figure 1F) and PDE5A expression showed that *MGMT* status (p=0,022) and PDE5A (p=0,0046) expression are independent prognostic factors in GBM. With respect to other clinical and biological characteristics, only EGFRvIII expression was inversely correlated with PDE5 positivity in GBM patients, as evaluated by Fisher exact test (p=0. 0306; HR 0.3230; 95% CI from 0.1209 to 0.8630). The extent of resection (total vs subtotal) did not influence the OS and did not correlate with PDE5 expression in the tumor mass.

**Figure 2:**
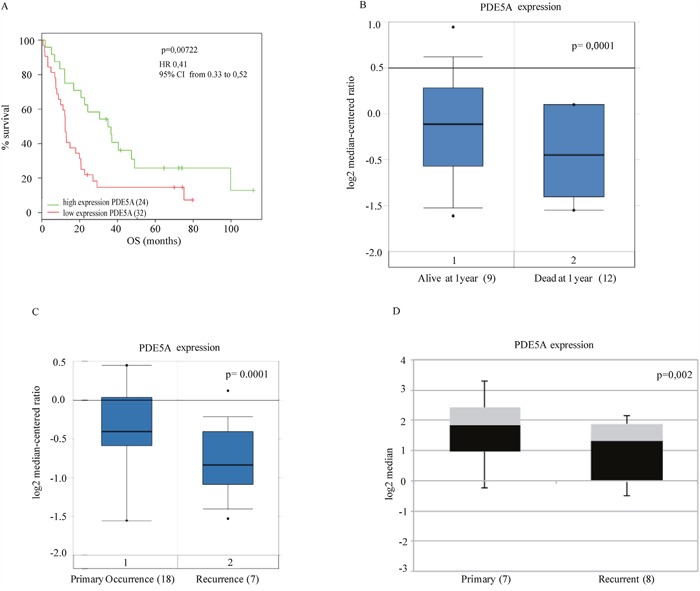
Clinical association of PDE5A gene expression with survival of GBM patients from public datasets **A**. The association between expression levels of PDE5A and OS was evaluated by Kaplan–Meier analysis **B**. and gene expression analysis to 1 year by box-plot graph, respectively in two public datasets [24, 25] (56 and 21 patients, respectively). Statistically significant results (*p*-value < 0.05) are indicated. The hazard ratios and 95% confidence intervals were calculated using a multivariable-adjusted Cox model, and the *p* values were calculated using a two-sided log-rank test. **C**. PDE5A expression levels from 25 GBM patients obtained from the public dataset of Liang et al. [24]. Casuistry samples have been divided between «primary occurrence» (18 patients) and «recurrence» (7 patients). PDE5A mRNA expression levels in these samples are reported in the box-plot. A statistically significant correlation is shown between PDE5 mRNA low levels and the GBM's relapse. **D**. PDE5A expression analysis of cell lines derived from primary (7) and recurrent glioblastomas (8) with resistance to O(6)-alkylating agents, BCNU and TMZ is shown as box plot graph. The expression values are processed by the public repository dataset of Bredel et al. [55].

We then assessed the OS in two different cohorts of GBM patients from publicly available gene expression datasets [24, 25]. As shown in Figure 2A and 2B we found that high PDE5 mRNA levels are significantly associated with a longer OS as compared to low PDE5 mRNA levels. In agreement, we also found that recurrent tumors showed lower PDE5 mRNA levels compared to primary tumors (Figure 2C). Moreover, performing an expression analysis of cell lines derived from primary and recurrent GBM with resistance to the O(6)-alkylating agents bis(chloroethyl)nitrosourea (BCNU) and TMZ [26], we found that cell lines obtained from recurrent gliomas showed lower PDE5 mRNA expression (Figure 2D). Altogether these results suggest that PDE5 expression plays a protective role in GBM.

### cGMP generating/hydrolytic pathways in GBM cell lines

To understand how PDE5 expression might affect GBM cells, we first analyzed its expression levels in five GBM cell lines and in a GBM stem cell line (line 83). Strikingly, we found that only T98G cells expressed high levels of PDE5, along with PDE1C, but not PDE1A and PDE3A, that represent other phosphodiesterases specific for cGMP hydrolysis (Supplementary Figure 1A-1D), while PDE6 and PDE11 were not expressed (not shown). PDE5 localized diffusely throughout the cell, but it was particularly concentrated in centrosomes [27] (Supplementary Figure 1B). We found that all the glioblastoma cell lines but A172 cell line expressed GUCY1A2, GUCY1A3, GUCY1B3, the subunits of the the dimeric soluble GC receptor activated by NO, as well as NPR2, the receptor-coupled GC isoform that binds C-type natriuretic peptide (CNP) (Supplementary Figure 1E). To understand if T98G cell line was responsive to these ligands, we stimulated the scrambled cells with 1μM S-nitrosoglutathione (GSNO, a synthetic nitric oxide donor) or 1μM type C natriuretic peptide (CNP) for 15 min. Either GSNO or CNP induced a consistent increase of intracellular cGMP levels (18 ± 3.6pmol/mg and 21±2.2pmol/mg, respectively) compared to control cells (2.35±0.6 pmol/mg), while sildenafil, slightly increased cGMP levels (3.5± 1.7pmol/mg) (Supplementary Figure 1F). Stimulation of T98G with both GSNO and sildenafil or with CNP and sildenafil strongly amplified the effect on cGMP levels (95±8.5pmol/mg and 102± 6.6pmol/mg, respectively). Moreover, sildenafil treatment alone was able to increase cGMP levels compared to the control (2.5±0.05pmol/mg and 80±3.5pmol/mg, respectively), after an overnight stimulation. Similarly to smooth muscle cells (S.D., unpublished observations) [28], PDE5 protein levels were up regulated in T98G by the presence of 5 μM MAP kinase inhibitors PD98059 or U0123, while they were down regulated by 10 ng/ml EGF. In the presence of both PD98059 and EGF, PDE5 levels were almost restored to the control levels (Supplementary Figure 1G, 1I). Interestingly, both GSNO and CNP independently induced Erk1/2 phosphorylation following 3 h of stimulation (Supplementary Figure 1H, 1L), suggesting that also PKG was activating the MAPK pathway.

### Effect of PDE5 knockdown on GBM cell growth, migration and invasiveness

**Figure 3:**
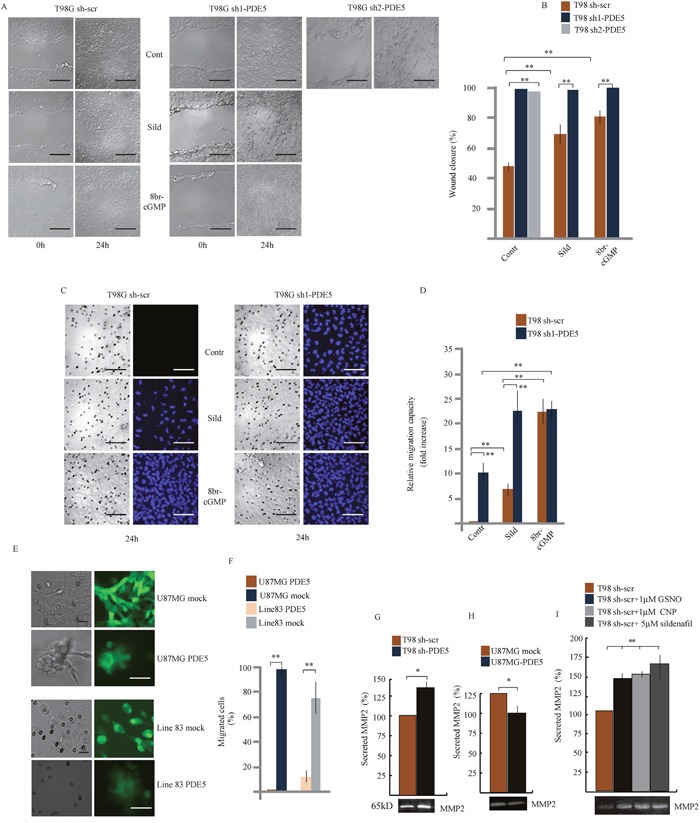
Effect of PDE5 knockdown or overexpression on cell migration and invasion *in vitro* **A**. Wound healing assay and **B**. Histogram showing the mean percentage ±SD of closure of sh-scrT98G or sh1 or sh2-PDE5 cells monolayers in the presence or absence of 1 μM sildenafil or 100 μM 8br-cGMP. **C**. Migration assay on matrigel cushions through transwell filters of sh-scr or sh1-PDE5 T98G cells in the presence or absence of 1 μM sildenafil or 100 μM 8br-cGMP. Scale bars= 50 μm. **D**. Histogram showing the relative migration capacity (fold increase ± SD) of cells that passed through the filter in the different treatments compared to control cells. **E**. Migration assay on matrigel cushions through transwell filters of mock- or PDE5-overexpressing U87MG (upper panels) or GBM line 83 cells (lower panels). Scale bar= 20 μm. **F**. Histogram showing the mean percentage ± SD of cells that passed through the filter. **G**. Histogram of the mean percentage ±SD of secreted MMP-2 in gelatin zymography (bottom image) in sh-scr or sh1-PDE5 T98G and **H**. in mock- or PDE5-overexpressing U87MG cells and **I**. in sh-scr T98G stimulated with CNP, GSNO or sildenafil at the indicated concentrations. Data have been obtained from three independent experiments. (*p < 0.05; **p < 0.001).

We chose T98G, U87MG and line 83 cells as *in vitro* models to silence or over-express PDE5 by using sh-PDE5 or over-expressing lentiviral particles, respectively (Supplementary Figure 2A-2B). As expected, cGMP levels were up-regulated after knockdown of PDE5 in T98G that have been silenced with two different sh-lentiviral particles (sh1 and sh2) and down-regulated in U87MG and line 83 cells overexpressing PDE5 (Supplementary Figure 2), confirming that modulation of PDE5 expression by these approaches was functionally relevant. MTS and H^3^-thymidine incorporation assays indicated that PDE5 knockdown or over-expression did not affect proliferation and survival of GBM cell lines (Supplementary Figure 2F-2H). To investigate if PDE5 expression affects GBM cell migration and invasiveness, we performed wound-healing and matrigel invasion assays in T98G cells. Wound-healing tests showed that sh1 or sh2-PDE5 T98G cells completely filled the gap area created by a scratch following 24 h of culture (100%), whereas control sh-scr cells only covered about 48.4% of the wound. Importantly, PDE5 inhibition with 1 μM sildenafil (70%) or 100 μM 8br-cGMP also increased the wound-healing ability of sh-scr cells (78%), whereas these agents did not affect the invasive behavior of cells that were already silenced for PDE5 (Figure 3A-3B).

**Figure 4:**
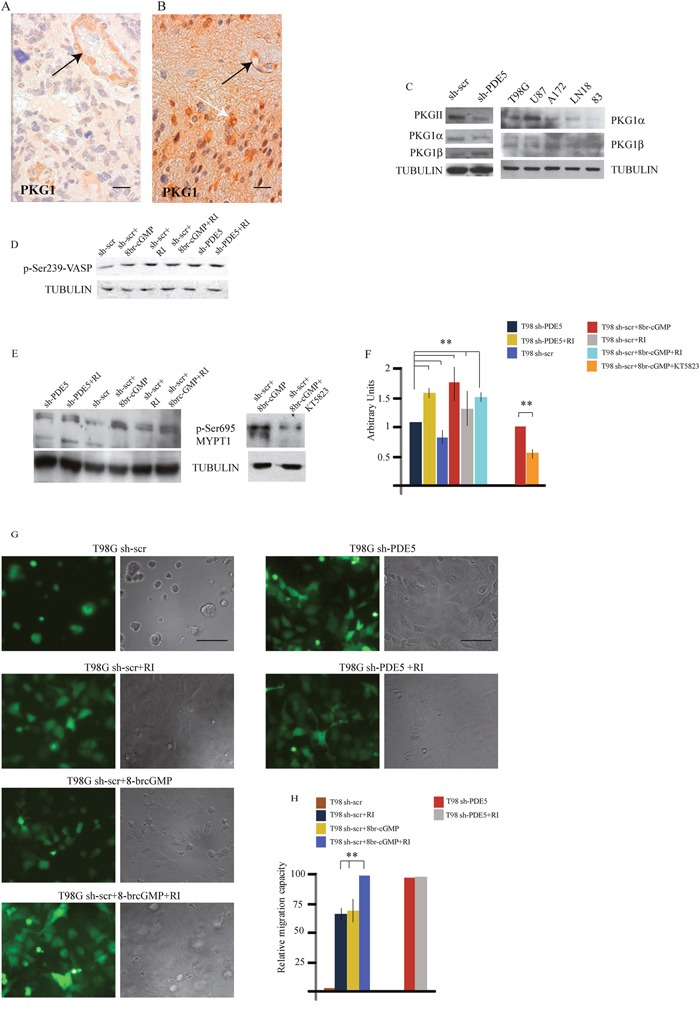
PKG expression and MYPT1 phosphorylation regulates GBM cells migration **A**. PKG1 low and **B**. PKG1 high GBM samples, as revealed by immuno-histochemistry. Black arrows point to PKG1 expression within the vessel walls. White arrow points to tumor cells. Scale bar=20μm. **C**. Expression levels of PKGII, PKG1α and PKG1β and total PKG1 in sh-scr and sh1-PDE5 T98G cells and in wtT98G, U87MG, A172, LN18, 83 GBM lines. **D**. pVASP levels in sh-scr or sh1-PDE5 T98G cells before and after Y-27632, 8br-cGMP (50 μM) treatments. Representative image out of 4 independent experiments. **E**. Phosphorylation levels of MYPT1 (pSer695) in sh-PDE5 or sh1-scr T98G upon the indicated treatments [8br-cGMP, 50 μM; Y-27632 (RI), 10 μM); KT5823 (PKGi), 2 μM]. **F**. Densitometric analysis of western blots from three independent experiments is shown. Bars represent the mean ±SD. **G**. sh-scr and sh1-PDE5 T98G invasion assays on matrigel cushions in the presence of Y-27632 or 8br-cGMP or both. **H**. Histogram showing the relative migration capacity (fold increase ± SD) of cells that reached the bottom of the matrigel cushion in the different treatments compared to control cells. (**p < 0.001).

Next, we tested whether PDE5 expression also affected GBM cell invasion. Sh-scr or sh1-PDE5 T98G cells were layered on top of matrigel cushions and the percentage of cells that passed through pores of filter membranes or reached the multiwell bottom was evaluated after 24 h (Figure 3C-3D and 4G, respectively). We observed that sh1-PDE5 cells were able to pass through the membrane pores, while sh-scr cells were always found on top of the matrigel cushion (10 fold increase in sh1-PDE5 *vs* sh-scr T98G cells). Sildenafil treatment strongly increased cell migration in both sh-scr or sh1-PDE5 T98G (5 fold increase and 2.2 fold increase *vs* untreated control, respectively). The increase in the number of migrating cells was even stronger after 8br-cGMP treatment (20 fold in sh-scr T98G cells *vs* untreated control and 2.1 fold in sh1-PDE5 T98G cells). In line with a negative role of PDE5 in the regulation of GBM cell migration, overexpression of PDE5 in PDE5-negative U87MG or GBM line 83 cells completely blocked their ability to migrate through the membrane after 24 h of culture (Figure 3E-3F). These results strongly indicate that high cGMP levels stimulate GBM cell motility and invasiveness.

To investigate whether the increased cell invasiveness correlated with metastasis-associated MMP secretion, the levels of MMP-2 and MMP-9 were measured in cell-culture supernatants from sh-scr or sh1-PDE5 T98G cells and from mock- or PDE5-overexpressing U87MG by gelatin zymography. We found that MMP2 levels were significantly induced in the supernatant of sh1-PDE5 T98G compared to sh-scr cells (Figure 3G; P=0.005) and reduced in U87MG overexpressing PDE5 compared to their controls (Figure 3H; P=0.005). We found that MMP-2 secretion by T98G sh-scr was also stimulated by other cGMP inducing agents such as CNP (1μM), GSNO (1μM) or sildenafil treatments (Figure 3I). MMP-9 was barely detectable in T98G cells in the conditions used to evaluate the activity of MMP-2, while we did not find any change in its levels in U87MG cells (not shown).

### PKG regulates GBM invasiveness

cGMP modulates cytoskeletal interactions through PKG activation [29, 30]. We then investigated if PKG1 was expressed in glioblastoma samples by IHC. Thirty GBM samples (15 with high and 15 with low or null PDE5 levels) were analyzed. We found that PKG1 was expressed in all the GBM samples (Figure 4A), although with different level of intensity, that were not significantly correlated to PDE5 expression. PKG1 was localized in glioblastoma cells and in the smooth muscle wall of tumor vessels. We found that PKG1α PKG1β and PKGII isoforms were all expressed in the different GBM cell lines. To understand if PKG activation by cGMP was involved in cell invasion, we performed invasion assays in the presence of the pan-PKG inhibitor KT5823 (2 μM). As expected, 8br-cGMP treatment or PDE5 knockdown stimulated T98G cell migration. Importantly, KT5823 was able to inhibit both basal and 8br-cGMP-induced cell motility in T98G sh-scr and in PDE5 silenced cells (Supplementary Figure 3). PKG phosphorylates Ser695 of the myosin targeting subunit of myosin phosphatase (MYPT1), the regulatory subunit of myosin light chain phosphatase (MLCP) [31]. PKG-mediated phosphorylation of MYPT1 blocks its subsequent inhibitory phosphorylation by Rho-kinase (ROCK) [32]. We found that pSer695 levels were increased in sh-PDE5 compared to sh-scr cells (Figure 4E) and in sh-scr cells treated with 100 μM 8br-cGMP. Conversely, inhibition of PKG by KT5823 reduced MYPT1 phosphorylation in cell treated with 8br-cGMP (Figure 4E), suggesting that the effect elicited by knockdown of PDE5 was mediated by PKG. Although KT5823 activity in inhibiting PKG has been questioned in intact cells [33], we found that it was effective in glioma cells, as also reported by Charles et al. [23].

Next, PKG activity was indirectly evaluated from the levels of phosphorylation of the vasodilator-stimulated phosphoprotein (p-VASP). In line with the hypothesis, we found that 8br-cGMP treated sh-scr T98G and that sh-PDE5 cells showed a stronger p-VASP band than sh-scr T98G cells, confirming that PKG activity was higher in these samples (Figure 4D). Since inhibition of the RHO/ROCK complex components can promote mesenchymal cell migration pattern through the regulation of acto-myosin contractility [34, 35], we treated sh-scr or sh-PDE5 T98G cells with 10 μM Y-27632 (a ROCK inhibitor, RI). We observed that, while all sh-scr cells were on top of matrigel, ~70% of cells were migrated toward the bottom of the plate after 24 h of incubation in the presence of 10 μM Y-27632 (Figure 4G-4H). In agreement with this observation, we found that ROCK inhibition induced a consistent increase of Ser695 p-MYPT1 levels in sh-scr T98G cells, whereas combined treatment with 8br-cGMP and Y-27632 did not increase it further (Figure 4E-4F). Notably, we found that ROCK inhibition also increased the levels of VASP phosphorylation (Figure 4D), suggesting that ROCK negatively regulates PKG in GBM cells. Furthermore, while 8br-cGMP induced about 70% of sh-scr-T98G migration through the matrigel cushion, combined treatment with Y-27632 was able to induce 100% of cell migration, suggesting that these agents synergistically promote GBM cell migration (Figure 4G-4H). Since sh-PDE5 cells already migrated to the bottom of the plate, addition of 10 μM Y-27632 did not further increase the migration rate of these cells (Figure 4G-4H). These results suggest that PDE5 might regulateGBM cell contractility and migration by balancing the activity between PKG and ROCK on MYPT1phosphorylation as well as on other substrates.

### PDE5 expression regulates sensitivity to ionizing radiations in GBM cell lines

**Figure 5:**
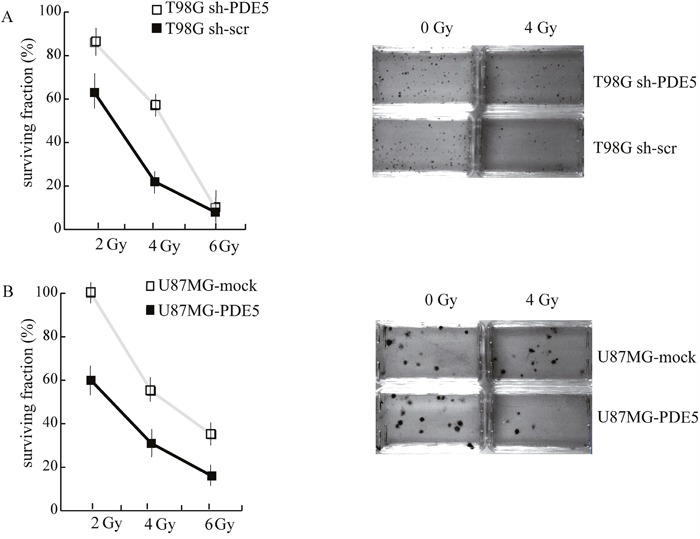
Effect of PDE5 on DNA repair and cell survival upon ionizing radiation **A**. Left: survival curves of sh-scr (black squares) or sh1-PDE5 T98G cells (empty squares) exposed to increasing X-ray doses from 2 to 6 Gy. Right: representative images of clonogenic assays in untreated control cells or in cells exposed to 4 Gy. **B**. Left: survival curves of PDE5-overexpressing (black squares) or mock-expressing U87MG cells (empty squares) exposed to increasing X-ray doses from 2 to 6 Gy. Right: representative images of clonogenic assays in untreated control cells or in cells exposed to 4Gy.

**Figure 6:**
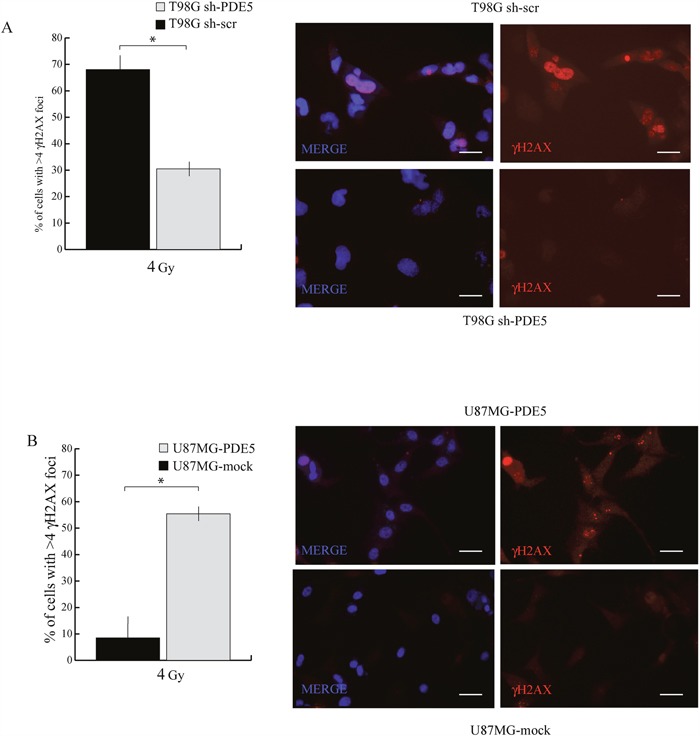
Effect of PDE5 expression on γ-H2AX foci induced by X-ray treatment **A**. Left: counts of nuclei containing ≥ 4 γ-H2AX foci in sh-scr or sh-PDE5 T98G cells; right representative immunofluorescence images of γ-H2AX foci. **B**. Left: counts of nuclei containing ≥ 4 γ-H2AX foci in mock- or PDE5-overexpressing U87MG cells; Right: representative immunofluorescence images of γ-H2AX foci. Cells were immunostained for γ-H2AX after 24 h at 4 Gy irradiation. (*p < 0.05; **p < 0.001). Bar= 10 μm.

Since all the patients analyzed in our study underwent radio- and chemotherapy treatments, the positive prognostic value of PDE5 expression may rely on the effect of this enzyme on GBM cells sensitivity to DNA-damaging agents. To test this hypothesis, sh-scr or sh1-PDE5 T98G and mock or PDE5 overexpressing U87MG cells were treated with X-ray doses from 2 to 6 Gy. Cell survival was assessed by colony forming assays after 14d of culture. We found that knockdown of PDE5 significantly increased the percentage of cells surviving to 2 to 4 Gy, while at 6 Gy few or no colonies were recovered from both cell types (Figure 5A). Conversely, PDE5-overexpressing U87MG cells showed a lower survival rate compared to mock-transduced cells exposed to radiation, even though the overall sensitivity to X-rays of U87MG was lower compared to T98G cells (Figure 5B). To understand if the increased survival of GBM cell lines lacking PDE5 was due to increased DNA damage repair, the kinetics of γH2AX staining were monitored in irradiated cells. We found that more than 90% of cells showed intense γH2AX staining within their nuclei 4 h post irradiation, regardless of PDE5 expression. However, 24 h post irradiation ~70% of sh-scr T98G cells showed nuclei containing more than 4 γH2AX positive foci/cell compared to 30% of sh-PDE5 cells (Figure 6A). Furthermore, PDE5-overexpressing U87MG cell also showed much higher percentage of γH2AX positive nuclei compared to mock-infected cells (Figure 6B). These results suggest that PDE5 expression impairs DNA-damage repair induced by ionizing radiations.

### PDE5 levels regulate PARP activity

**Figure 7:**
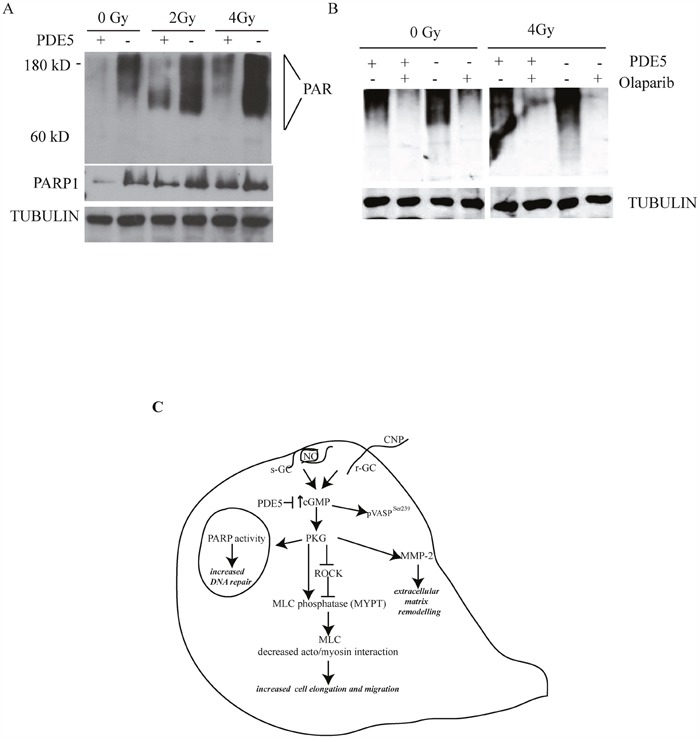
PDE5 knockdown increases PARP1 activity in GBM cells **A**. sh-scr (PDE5+) or sh1-PDE5 (PDE5-) T98G cells were treated with increasing X-ray doses from 2 to 4 Gy and probed with anti-PAR and anti-PARP-1 antibodies. **B**. sh-scr or sh1-PDE5 T98G cells were exposed to X-rays at 4 Gy in the presence or absence (+/-) of the PARP1 inhibitor olaparib (1 μM). Total protein PARylation levels were assayed after 24 h. **C**. Schematic representation of cGMP signaling pathway promoting migration and DNA repair in GBM cells.

It has been recently reported that PDE5 inhibition protects hair cells and spiral ganglion neurons from sound-induced cell damage by increasing poly (ADP-ribose) polymerase (PARP) activity [36]. To understand if a similar mechanism was activated in GBM cells, we evaluated the levels of protein poly(ADP-ribosyl)ation (PARylation) in sh-scr and sh1-PDE5 T98G cells before and after different doses of X-ray exposure. In the absence of irradiation, sh1-PDE5 T98G cells showed higher protein PARylation levels (detected with the anti-PAR antibody as a smear) and higher PARP-1 expression compared to sh-scr cells. Moreover, PARylation strongly increased following X-ray exposure and such increase was higher in sh-PDE5 compared to sh-scr T98G cells (Figure 7A). Treatment with the PARP inhibitor olaparib (1 μM) strongly reduced PARylation in T98G (Figure 7B) and reduced sh-PDE5 cell viability after exposure to X-rays (not shown). These data indicate the involvement of PARP activity in radio-resistance of GBM cells and suggest that its pharmacological inhibition might counteract aggressiveness of PDE-negative GBM cells.

## DISCUSSION

PDE5 finely regulates intracellular cGMP levels generated by GCs. These enzymes are directly activated by a range of ligands such as NO and natriuretic peptide that bind s- or p-GC isoforms, respectively [37]. The role of the NO/GC/cGMP/PDE5 axis in tumor biology has been extensively studied, however its precise role has not been unequivocally established. Although genetic alterations of the *PDE5* locus are relatively rare, increased PDE5 expression was shown to play a role in tumorigenesis in a variety of epithelial cancers [38, 39] and to be correlated with OS in breast cancer [40]. By contrast, PDE5 expression inversely correlates with tumor invasiveness in metastatic melanoma [19].

Our study first aimed at evaluating the association between PDE5 expression and clinical outcome in GBM patients. In three independent cohorts of GBM patients we found that high PDE5 protein and mRNA levels in GBM cells significantly associate with less aggressive cancer behavior and longer OS. Moreover, multivariate analysis identified PDE5 as a strong independent prognostic factor in GBM. Thus, our study indicates PDE5 expression as an important index of favorable prognosis in this disease. To elucidate the molecular mechanisms underlying the role of PDE5 in GBM, we genetically engineered GBM cell lines to stably express high or low levels of PDE5. Using these cell models, we unveiled two distinct roles of PDE5 in attenuating GBM aggressiveness: 1) decreased tumor cell invasion and migration via stabilization of acto-myosin interactions and decreased MMP-2 secretion; 2) enhanced radio-sensitivity by inhibiting DNA repair. The molecular players of NO/GC/cGMP/PDE5 axis were expressed in all the GBM cell lines analyzed and activation of this axis in T98G resulted in a significant increase of cGMP levels. The mechanisms underlying the reduced invasiveness of PDE5 expressing tumors correlated with low cGMP levels, as PDE5 silencing or 8br-cGMP treatment induced an invasive phenotype. Our data are in apparent contrast with recent reports describing a cytotoxic effect of PDE5 inhibitors in *in vitro* treated brain cancer cell lines [16–18, 41]. However, our *in vitro* studies confirmed that PDE5 inhibition or cGMP treatment acted to favour GBM aggressiveness and invasivity. Moreover, in line with our data, a role of cGMP in promoting murine and human melanoma cell growth and invasion has been recently reported [20], suggesting that PDE5 expression may play a protective role in cancer types of neuro-ectodermal origin.

Indeed, as in melanoma cells, ERK1/2 phosphorylation was induced by increased cGMP levels, as it occurs following GSNO or CNP stimulation, suggesting that migration of stimulated cells could in part be mediated by the increase of MAPK activity. High cGMP levels induced phosphorylation of MYPT1 at Ser695, an event that decreases actin/myosin interactions and promotes relaxation and forward motility in fibroblasts [42]. Although migrating cells activate Rho GTPases to control myosin phosphorylation and cell motility, they also control GC activity and cGMP increase [43]. Indeed, GBM invasiveness has been shown to be strongly induced by negative regulation of ROCK2 activity [44–46]. We demonstrated that ROCK inhibition increased GBM invasiveness and increased Ser695 MYPT1 levels, similarly to what has been described for rat neuronal migration [47]. Although ROCK inhibition and 8br-cGMP stimulation synergistically increased matrix invasion, Ser695 p-MYPT1 levels did not further increase, suggesting that MYPT1 phosphorylation was not the only event following ROCK inhibition or PKG activation leading to the control cancer cell migration. In line with these findings, PKG activation was shown to promote breast cancer cell invasion via caldesmon phosphorylation and inhibition of actin/myosin interactions [48]. The decreased invasiveness of PDE5-overexpressing GBM cells was associated with lower MMP-2 levels, while cGMP elevating agents such as GSNO, CNP or sildenafil up-regulated MMP-2 secretion in T98G cells, as previously shown in synovial cells, colon cancer cells and NO-stimulated murine mammary adenocarcinoma cells [49, 50]. The finding that sildenafil alone increased MMP-2 secretion by T98G cells after an overnight incubation is in line with its effects on glioblastoma cell migration and is similar to the effects induced by PDE5 silencing, suggesting that continuous PDE5 inhibition might stimulate GC activity in tumor cells, as it was shown by the increase of cGMP levels. Our results suggest that GBM cell invasiveness can be modulated by PDE5 expression and ROCK activity that, by inhibiting PKG, controls cytoskeletal interactions and extracellular matrix remodeling. Indeed, PKG1 was found to be expressed in all the GBM samples, although at different levels, suggesting that NO/GC/PKG pathway activation can act on tumors to promote aggressive potential. PDE5-expressing GBM cells showed defective DNA repair activity and reduced cell survival to irradiation compared to PDE5-low or -negative cells. Moreover, high cGMP levels induced resistance to DNA damaging agents through increased DNA repair proficiency (see Figure 7C). Since all the GBM patients in our study also underwent radio- and chemotherapy, our results strongly suggest that the increased OS observed in high PDE5 patients is linked to enhanced sensitivity of GBM cells to DNA damage. The increased repair mechanism in sh-PDE5 cells correlated with increased PARP-1 activity and this effect was reversed by the presence of the PARP inhibitor olaparib. In agreement with these results a previous report demonstrated that activation of cGMP-PKGI signaling pathway through PDE5 inhibition culminates in the activation of PARP-1 in hair cells and in the spiral ganglion [36].

In conclusion, our results indicate that PDE5 expression lowers the invasive potential and DNA repair ability of GBM cells, explaining the poor survival in PDE5-low GBM patients. Stratification of GBM into PDE5-low and -high expression groups may allow identification of tumors that are more invasive and resistant to ionizing radiations. This implies that PDE5-negative GBM patients could be predicted to be resistant to radiotherapy and preferably enrolled in alternative therapeutic procedures, such as combined treatment with PARP inhibitors.

PDE5 inhibitors (PDE5i) have been proposed to enhance anti-tumor efficacy of chemotherapeutic agents by increasing permeability of the brain blood barrier [17]. Potential PDE5i therapies would then be applicable only to PDE5-negative GBMs, that would be insensitive to these drugs, but not to PDE5-positive GBMs. Moreover, the use of PDE5i in erectile dysfunction as well as in pulmonary hypertension should be evaluated in PDE5 positive GBM patients. We propose PDE5 as a highly statistically significant prognostic marker of increased OS in GBM patients and identify PKG, ROCK and PARP1 as potential therapeutic targets for this subset of brain cancers.

## MATERIALS AND METHODS

Sixty-nine tumor tissue samples were collected at surgery from patients who underwent craniotomy for complete or partial resection of GBM at the Institute of Neurosurgery, Catholic University School of Medicine, in Rome. Patients were eligible for the study if a diagnosis of GBM was established histologically by the neuropathologist in accordance with the WHO classification [2]. The patients were 27 to 80 year old at the time of diagnosis (median age, 62 yrs); 45 were men and 24 were women. In all cases, the tumor samples were obtained by resection before treatment with radiation and TMZ. Thirteen out 69 patients (18,8%) underwent to subtotal tumor resection. After surgery, the patients received radiotherapy to limited fields (2 Gy per fraction, once a day, 5 days a week, 60 Gy total dose) and concomitant TMZ (75 mg per square meter of body surface area per day) for 7 days a week from the first to the last day of radiotherapy followed by six cycles of adjuvant TMZ (at 150-200 mg per square meter of body surface area on days 1 to 5) given at 4 week intervals. Survival was calculated from the date of surgery when a diagnosis of GBM had been established. The disease was considered to have progressed if the sum of products of perpendicular diameters of any enhancing lesion increased by 25% of initial measurements, if a new lesion was evident on axial contrast-enhanced T1-weighted magnetic resonance imaging scan, or if the patient's neurologic condition worsened and required an increased dose of steroids [51]. Only those patients who completed radiation therapy and concomitant TMZ were included. Patients who died during cycles of adjuvant TMZ were included. Immunohistochemical patterns were assessed as previously reported [52, 53]. The Ki-67 (1:1000, MIB-1, Dako, Milan, Italy) labeling index was defined as the percentage of positive nuclei of a total of 2000 tumor cells counted using an eyepiece grid. The positive nuclei were counted by 2 pathologists (L.M.L. and M.M.). In order to minimize contamination by normal cells, the tumor areas dissected for DNA and mRNA extraction contained at least 80% of tumor cells. Expression of EGFRVIII was performed as previously described [9]. The O(6)-methylguanine-DNA methyltransferase (MGMT) promoter methylation patterns were studied by methylation-specific PCR using primers specific for methylated and unmethylated DNA on genomic DNA extracted from paraffin-embedded tissue (QIAmp DNA Mini kit, Qiagen, Milan, Italy) [54]. The annealing temperature was 60°C. DNA from normal lymphocytes treated or untreated with SssI methyltransferase (New England Biolabs, Ipswich, MA, USA) was obtained for positive and negative controls, respectively. PCR products were separated onto 3% agarose gel, stained with ethidium bromide, and visualized under UV illumination.

For mRNA expression data, we used the publicly available data sets: GSE2221 [55] or in [24, 25]. The data sets were analyzed by the Oncomine platform [26].

### RT-PCR

Total RNA from the different cell lines was extracted with Trizol reagent and treated with DNase I to avoid potential contamination by genomic DNA. DNA-free RNA was reverse transcribed using Bioline (BIO-65043) tetro cDNA Synthesis Kit according to the manufacturer's instructions.

For semi quantitative RT-PCR 25 cycles were performed for the amplification of Actin, 35 cycles for for all the others. Primers for cDNA amplification were: GUCY1A2 for 5′GGTTACAGGGATGCAGAAAAGAAT3′; rev 5′TTCA GGGAGCTCTTTGCATAGG3′. NPR2 for 5′ACCATTAT CGTACCCTGGTT3′, rev 5′ TCTCGGTACGTGATCAC CAATA3′. PDE1A for 5′AGCAATGGTCTTTGCTGCTG; rev 5′AGTCGATAAGCTGCACTCAC. PDE3A for 5′CGT CACCTTCGCTAGTGAAA; rev 5′AACTCGTCTCAACA AGCCAG. ACTIN for 5′GCGAGAAGATGACCCA GATCA ; rev 5′CACAGGACTCCATGCCCAGGA. GUCY1B3 for 5′ GAGGAGTACAVACTAGGTTCCAGT; GUCY1B3 rev 5′ CACATGAAGCTCACATCATC; GUCY1A3 for AGACAGTAGACCTTCTGTGCTC; GUCY1A3 rev TCCACCTTGTAGACATCCAG.

### Statistical analysis

OS obtained from patients herein analyzed was calculated from the date of surgery when a diagnosis of GBM was established, to death from any cause. Progression-free survival (PFS) was determined from the date of surgery until progression or death. Survival curves were plotted using the Kaplan-Meier method and differences between groups were evaluated using the log-rank test. Multivariate analysis for survival was performed by using the Cox proportional hazards model to control for age, Karnofsky performance status (KPS), Ki67 measurement values, MGMT promoter methylation, expression of EGFRvIII, surgical resection (subtotal or total) and PDE5A expression. Continuous variables not normally distributed are reported as median and 95% CI. Comparison of continuous variables not normally distributed was performed using the Mann-Whitney U test; continuous variables normally distributed are reported as mean and standard deviation (SD). Comparison of continuous variables normally distributed was performed using the Student's t test. Comparison of categorical variables between the two groups was performed by the Chi-square statistic, or using the Fisher exact test when appropriate. All p-values are based on Student's t two-tailed tests and differences were considered statistically significant when p<0.05. Asterisks indicate the level of statistical significance (*p < 0.05; **p < 0.001).

OS obtained from GEO dataset ID: GSE13041 [25] was evaluated by using Kaplan-Meier analysis and Cox proportional hazard regression model. Samples were sorted by the intensity level and divided in two subgroups (high intensity level, low intensity level). Approximately the same amount of samples were used for comparison in the two groups where the difference in the signal level was wider (high intensity *vs* low intensity). The log-rank test was used to evaluate differences between curves.

### Immunohistochemistry for PDE5A, PKG1 and SMA

Formalin-fixed, paraffin embedded sections (4μm thick) were mounted on positive charged glass slides. For PDE5A and PKG detection, deparaffinized and rehydrated sections were retrieved in TE solution (pH 9). Endogenous peroxidase was blocked (ScyTek, Logan, UT) and then sections were incubated for 1h at room temperature with rabbit polyclonal antibody anti-PDE5A (1:100 diluition, H-120, sc 32884; Santa Cruz Biotech, Heidelberg, Germany) or with rabbit polyclonal anti-PKG (1:100 dilution, ADI-KAP-PK005; Enzo Life Sciences, Italy) followed by the avidin-biotin-peroxidase complex method (UltraTek HRP Anti-polyvalent, ScyTek). 3,3′ diaminobenzidine was used as the enzyme substrate to observe the specific antibody localization, and Mayer hematoxylin was used as a nuclear counterstain. Normal central nervous system biopsies were used as internal control. Tumor sections stained in the absence of the primary antibody were used as negative controls. For SMA staining, tissue sections were incubated with anti-α-smooth muscle actin-FITC (clone 1A4, Sigma-Aldrich, 1:100) and nuclei counterstained with Hoechst. Immunofluorescence was evaluated by epifluorescence using Leica CTR6000 microscope. All samples were stained more than once and the results were highly reproducible.

### Cell culture, proliferation and viability assays

The GBM cell lines (T98G, U87MG, A172, U251, LN18) used in this study were grown in DMEM, (Euroclone; Milan, Italy) with 10% fetal bovine serum (FBS) (Euroclone). T98G and U87MG cell proliferation was tested using ^3^H-thymidine incorporation assay [56] and cell viability was tested by MTS colorimetric assay (Promega, Milan, Italy). Glioblastoma stem cell line 83 was derived from patient derived glioblastoma obtained after informed consent and surgery, validated and grown in stem cell medium as previously reported [57]. The MAPK inhibitors PD98059 and U0126 were from Calbiochem (Milan, Italy). hEGF and hbFGF were from Immunological Sciences (Rome, Italy). Olaparib was obtained from Selleckchem (Milan, Italy). S-nitrosoglutathione (GSNO, a synthetic nitric oxide donor) and type C natriuretic peptide (CNP) were from Sigma-Aldrich (Milan, Italy).

### Migration and invasion assays

For wound healing assays, 1×10^5^ cells per well were seeded in 24-well culture plates to obtain a confluent monolayer. Confluent monolayers were then scored with a sterile pipette tip to make a scratch of approximately 0.4–0.5 mm in width. Culture medium with any dislodged cells was then removed and cells were treated with sildenafil or 8br-cGMP (a non-hydrolysable cell-permeable analog of cGMP) (Sigma-Aldrich). Cells were allowed to migrate through the scratched area for 24 hours. Cell migration was visualized using Leica CTR6000 microscope. The wound area was measured by the program Image J software (NIH, Bethesda, MD). The percentage of wound closure was estimated by the following equation: wound closure % = [1 − (wound area at *T*t/wound area at *T*o)]x100%, where *T*t is the time after wounding and *T*o is the time immediately after wounding. Three independent experiments were performed. The *in vitro* cancer cell invasion assays were performed in 24-well transwell insert with 8μm pore size coated with 50 μL matrigel (Corning Life Sciences, Sigma-Aldrich). Two × 10^4^ cells were added to the upper chamber in 0.2 mL serum-free medium; the bottom chamber contained medium with 10% FBS which acted as cell attractant. For migration of GBM line 83, cells were directly seeded on top of matrigel in serum free medium and the bottom chamber contained 10 ng/ml bFGF and 10 ng/ml EGF supplemented medium. After 24 hours incubation, cells that reached the underside of the filter were stained with Hoechst 3342 dye (Sigma-Aldrich) and counted for Hoechst or EGFP fluorescence by fluorescent microscopy based on five-field digital images taken randomly at 200× magnification. Three independent experiments were performed. In some experiments we modified the transwell invasion assay by seeding 10^3^ cells in serum free medium on top of a 50 μl matrigel cushion in 96 well chambers. After 24 h, the ratio of the number of cells at the bottom of the well on the total number of cells was obtained.

### Colony assay after DNA damaging treatments

For irradiation experiments, exponentially growing sh1-PDE5 and sh-scr T98G or mock and PDE5 overexpressing U87MG cells were seeded at 20/cm^2^ density and exposed to 2,4,6 Gy dose of acute ^137^Cs gamma irradiation, at a dose rate of 0.8 Gy/min (Gammacell 40 Exactor Best Theratronics at the ISS, Rome, Italy). To follow recovery after DNA damage, cells were then incubated at 37°C for 14 days (T98G cells) or 21 days (U87MG cells).

Surviving colonies were fixed with 100% methanol and then stained with Giemsa staining (Sigma-Aldrich) for 1 hour. Colonies ≥0.5 mm in diameter, were counted using a light microscope (Nikon, Milan, Italy). Four independent experiments were performed for each treatment.

### Immunofluorescence analysis

Cells grown on glass coverslips after mock or 4-Gy irradiation were fixed with 4% paraformaldehyde and incubated with rabbit anti-γ-H2AX antibody (Millipore, Milan, Italy) and Alexa Fluor® 594-conjugated secondary antibody (Thermofisher, Milan, Italy). T98G grown on glass coverslips were also incubated in anti PDE5 antibody and in Alexa Fluor® 594-conjugated secondary antibody. U87MG cells were used to validate anti-PDE5 antibody specificity (not shown). Nuclei were counterstained with Hoechst 33349. For each sample >200 cells, containing four or more γ-H2AX foci/nucleus, were scored as positive. Three independent experiments were performed was performed on T98G.

### List of WB antibodies

Anti-tubulin mouse monoclonal (A2066, Sigma-Aldrich 1:1000), anti PKGIα goat polyclonal (1:1000,N-16, sc10335 Santacruz Biotech), anti-PKGIβ goat polyclonal (1:1000, L-16, sc10341), anti PKGII (1:1000, T-10, sc10346, goat polyclonal, SantaCruz Biotech), anti pSer239-VASP mouse monoclonal (1:1000, 16C2, sc101439, SantaCruz Biotech), anti p- MYPT1 (Ser695) rabbit polyclonal (1:1000, A-6, sc-33360 Santacruz Biotech), anti-PAR mouse monoclonal (1:1000, Trevigen, Gaithersburg, MD, USA), anti-PARP-1 rabbit polyclonal (1:1000, SantaCruz Biotech), anti PDE1C rabbit polyclonal (1:1000 Fabgennix, TX, USA). Densitometric analyses of western blots were performed by ImageJ (http://rsb.info.nih.gov/ij/index.html).

### Lentiviral vector production and transduction

Lentiviral PDE5 silencing vectors [RHS4430-101103886 Clone V3LHS_325328 (silencing sequence: CGGTTAATGCAGAAGTTG) sh1PDE5] and lentiviral scrambled control [RHS2349] were purchased from Dharmacon (Milan,Italy). Another PDE5 ORF region was chosen to prepare a second sh-lentiviral construct (sh2) using cagaagaatggtacaaatcca as silencing sequence and cloned in pLKO plasmid (EcoRI-AgeI) (Addgene). For PDE5 overexpression human PDE5 A1 ORF was cloned into pRRLsin.PPT.CMV.NTRiresGFPpre lentiviral vector in the Xba5′-Xho3′ sites. All the lentiviral particles were obtained in Hek293T cells [58] and supernatant was concentrated by ultracentrifugation at 28,000 rpm for 2 h at 4°C. Cells were infected at MOI 10 and stable pools of cells were selected with 5 μg/mL puromycin.

### Gelatin zymography analysis

Conditioned media from sh1-PDE5, sh-scr T98G or from mock and PDE5 overexpressing U87MG cells at 72 hours of culture were used for the detection of MMP-2 and MMP-9 by gelatin zymography analysis as described in [59]. Conditioned media from sh-scr T98G, sh-scr T98G stimulated with GSNO, with CNP or with sildenafil were collected after 24h of stimulation. Quantification of MMP-2 and MMP-9 and western blot bands were achieved after image capture and by computerized image analysis using image J software. The secretion of MMP-2 in mock-treated cells was taken as 100%. Three independent experiments were performed.

### cGMP measurement

The cGMP levels in the glioblastoma cell lines were measured using an ELISA kit according to the manufacturer's instructions (ADI-900-014, Enzo Life Sciences, Italy). cGMP concentration was normalized per mg of total proteins.

## SUPPLEMENTARY FIGURES


