# Supplementary material for: Type 5 phosphodiesterase regulates glioblastoma multiforme aggressiveness and clinical outcome
Source: Oncotarget. 2017 Jan 14;8(8):13223–39. doi: 10.18632/oncotarget.14656 (PMC5355091; doi:10.18632/oncotarget.14656)
Supplement: Supplementary file 1 [file oncotarget-08-13223-s001.pdf]

Type 5 phosphodiesterase regulates glioblastoma multiforme aggressiveness and clinical outcome

SUPPLEMENTARY FIGURES

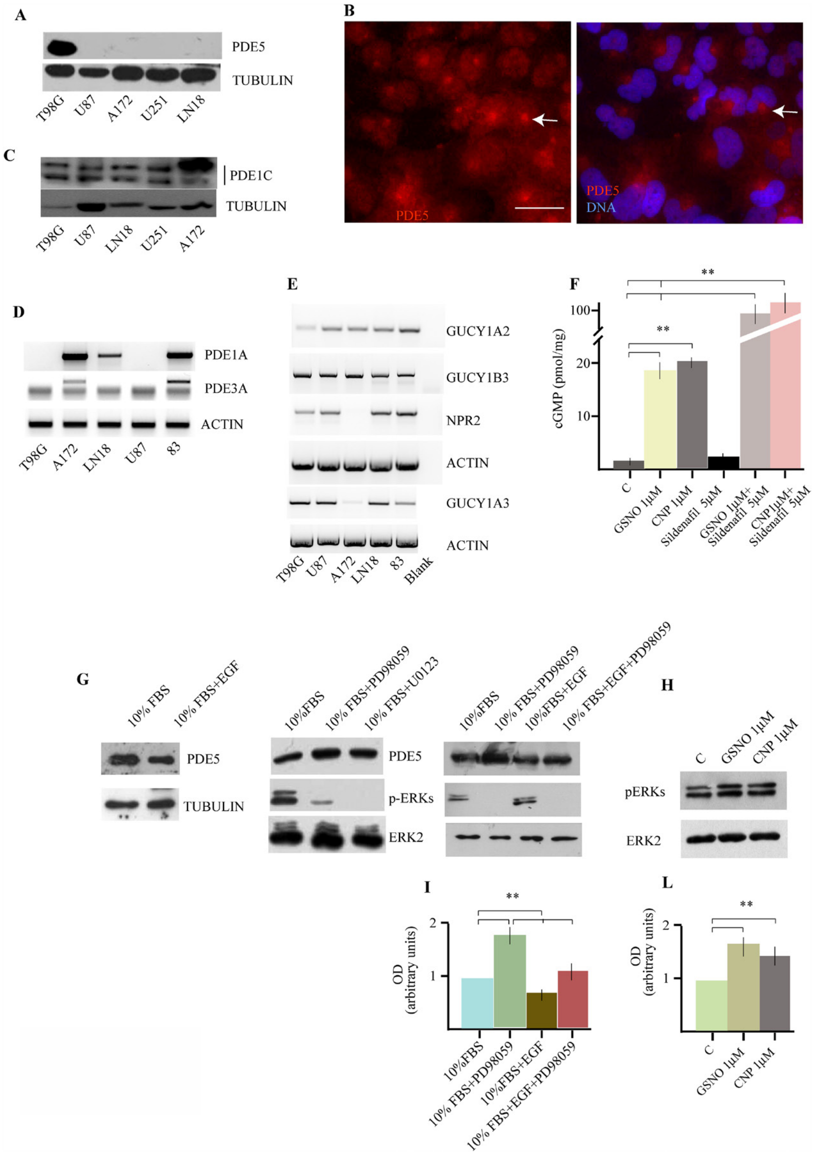

Supplementary Figure 1: Expression of cGMP-generators and cGMP/PDEs in GBM cell lines. A. Western blotting analysis showing PDE5 expression in T98G, U87MG, A172, U251, LN18 GBM cell lines; **B.** PDE5 subcellular localization in T98G cell line by immunofluorescence analysis. Bar= 10 µm. **C.** Expression of PDE1C by western blot and **D.** expression of PDE1A and PDE3A by semiquantitative rt-PCR in the glioblastoma cell lines. **E.** Expression of GUCY1A2, GUCY1A3, GUCY1B3 and NPR2 by semiquantitative rt-PCR in glioblastoma cell lines **F.** cGMP intracellular levels (ELISA) in scrambled T98G stimulated with the indicated drugs for 15 min obtained from three independent experiments. Bars represent the mean±SD. **G.** Regulation of PDE5 levels by MAPK activated pathway (EGF, left panel) and by MAPK inhibitors (PD98059 and U0123, middle and left panels). Experiments were performed after an overnight stimulation in the continuous presence of the corresponding factors. **H.** MAPK activated pathway by cGMP inducing agents in T98G cells. Experiments were performed after 15 min stimulation with the corresponding factors. **I.** Histogram of the densitometric analysis of western blots from five independent experiments. Bars represent the mean ±SD. **L.** Histogram of the densitometric analysis of western blots from four independent experiments. Bars represent the mean ±SD.

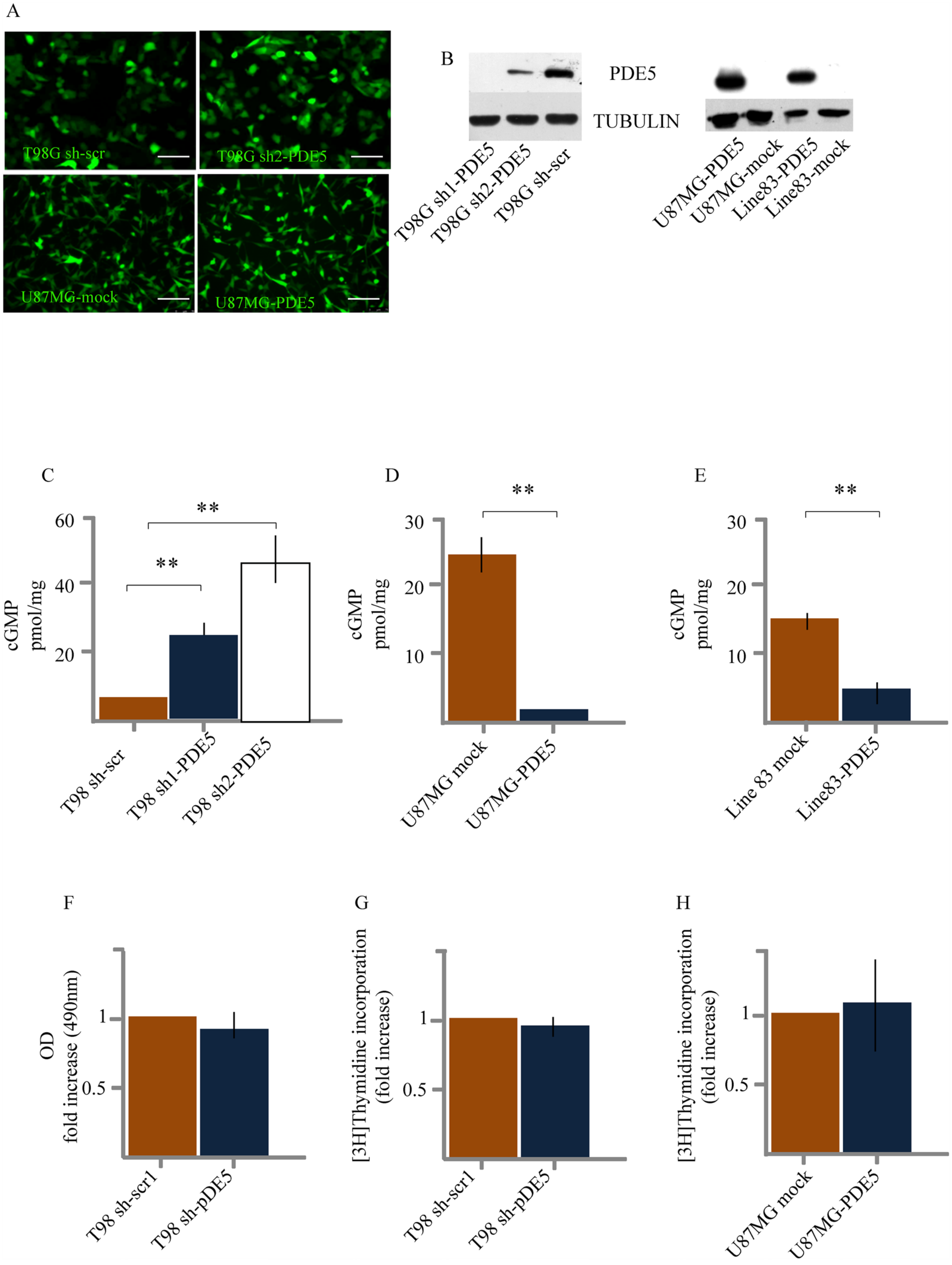

Supplementary Figure 2: Effect of PDE5 knockdown and overexpression on cell viability, proliferation and cGMP regulation. A. Enhanced Green Fluorescent Protein (EGFP) expression **B.** PDE5 protein levels cGMP intracellular levels (ELISA) **C-E.**, cell viability (MTS colorimetric assay 490 nm) **F.** and cell proliferation ([^{3}H]-thymidine incorporation assay) **G-H.** in: sh-scr (brown bar), sh1- (black bar) or sh2- (white bar) PDE5 T98G and in mock- or PDE5-overexpressing U87MG cells and in GBM stem cell line 83. Bar= 50 µm.

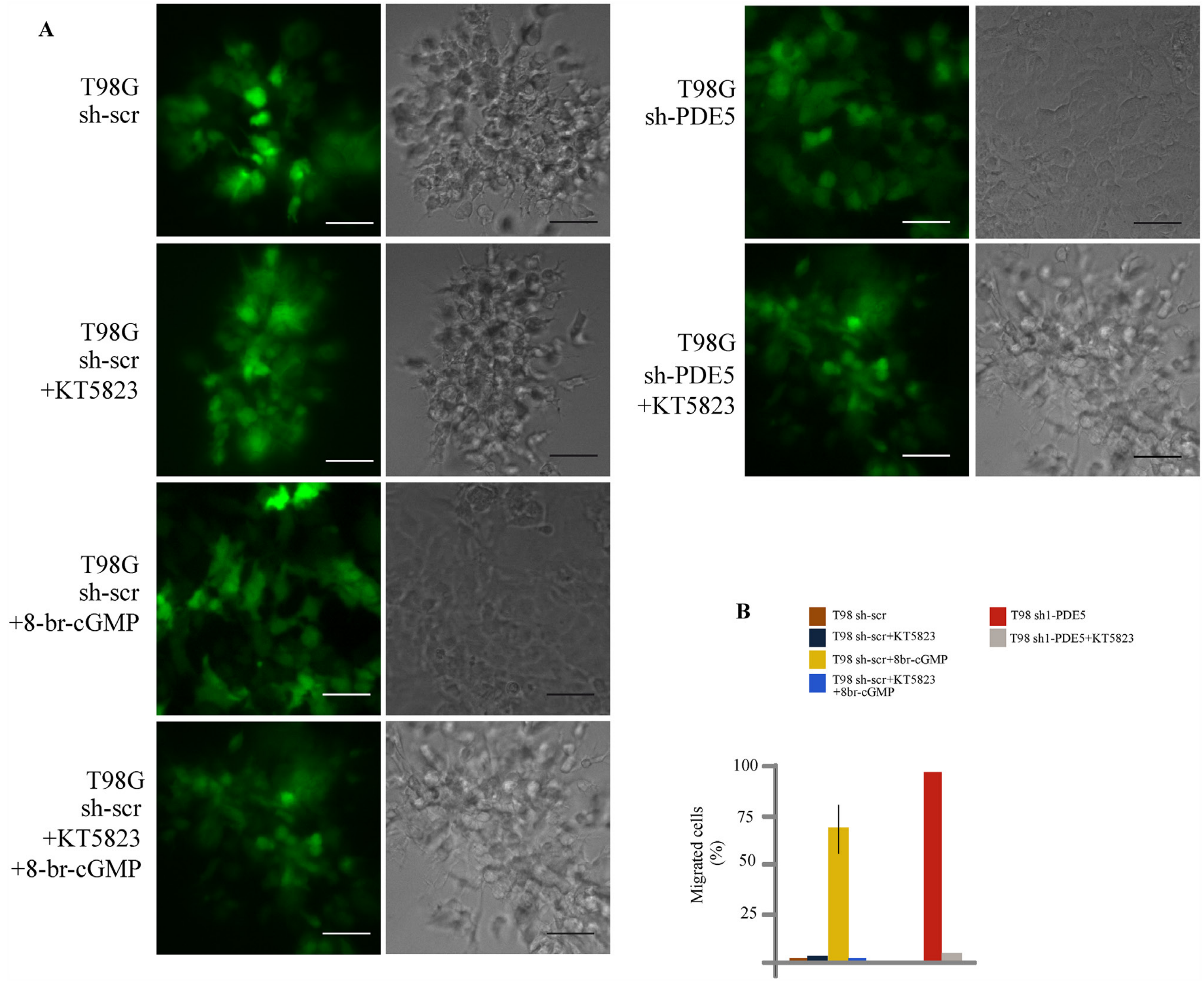

Supplementary Figure 3: Effect of PKG activation on GBM cells invasion ability. A. Sh-scr or sh1-PDE5 T98G cells were seeded on top of a matrigel cushion and incubated overnight in the presence or absence of the pan-PKG inhibitor KT5823 (2 µM), 8br-cGMP (only T98G sh-scr cells) (100 µM) or both. Bar= 50 µm. **B.** Histogram representing the results of three independent migration assays.
